# Solution-Processable NiO_x_:PMMA Hole Transport Layer for Efficient and Stable Inverted Organic Solar Cells

**DOI:** 10.3390/polym15081875

**Published:** 2023-04-14

**Authors:** Tianyu Kong, Genjie Yang, Pu Fan, Junsheng Yu

**Affiliations:** State Key Laboratory of Electronic Thin Films and Integrated Devices, School of Optoelectronic Science and Engineering, University of Electronic Science and Technology of China, Chengdu 610054, China; ty_kong@std.uestc.edu.cn (T.K.); genjieyang@std.uestc.edu.cn (G.Y.)

**Keywords:** inverted organic solar cell, hole transport layer, solution, nickel oxide, nanoparticle, poly(methyl methacrylate)

## Abstract

For organic solar cells (OSCs), nickel oxide (NiO_x_) is a potential candidate as the hole transport layer (HTL) material. However, due to the interfacial wettability mismatch, developing solution-based fabrication methods of the NiO_x_ HTL is challenging for OSCs with inverted device structures. In this work, by using N, N-dimethylformamide (DMF) to dissolve poly(methyl methacrylate) (PMMA), the polymer is successfully incorporated into the NiO_x_ nanoparticle (NP) dispersions to modify the solution-processable HTL of the inverted OSCs. Benefiting from the improvements of electrical and surface properties, the inverted PM6:Y6 OSCs based on the PMMA-doped NiO_x_ NP HTL achieves an enhanced power conversion efficiency of 15.11% as well as improved performance stability in ambient conditions. The results demonstrated a viable approach to realize efficient and stable inverted OSCs by tuning the solution-processable HTL.

## 1. Introduction

In recent years, organic solar cells (OSCs) based on non-fullerene acceptors (NFAs) have continued to set new efficiency records by virtue of developments in novel organic photovoltaic materials as well as innovations in device-processing techniques [1,2,3,4]. For the future commercialization of NFA-based OSCs, in addition to realizing the highest possible device efficiency, it is also crucial to achieve highly stable OSCs via facile large-scale, roll-to-roll compatible fabrication processes [5,6,7,8]. A modern single-junction OSC device typically consists of a bulk-heterojunction (BHJ) active layer, top metal electrodes, bottom transparent electrodes, and charge transport layers between the active layer and the electrodes. For the conventional-structured devices, metals of low work function (WF), namely aluminum (Al), calcium (Ca), or barium (Ba), are used as the cathodes on the top. In the inverted device structure, the direction of charge carrier transport is reversed. Therefore, silver (Ag) or gold (Au) with high WF are utilized as the top anodes. As Ag and Au are stable metals under ambient conditions, OSCs with the inverted structure are generally considered to possess higher stability than the conventional-structured devices; hence, they are more favorable for the mass production of practical OSCs [9,10].

Charge transport layers are introduced to improve the interfacial contacts between the active layer and electrodes and to enhance the electron/hole extraction efficiencies. For inverted OSCs, the electron transport layer (ETL) is fabricated on the transparent metal oxide cathode that can withstand high temperatures. In contrast, the hole transport layer (HTL) of the inverted device has to be deposited atop the BHJ active layer, which is vulnerable to excessive heat, moisture, and chemicals [11,12,13,14]. For such reasons, the materials and fabrication processes of the HTL in inverted OSCs demand careful selections to minimize the impacts on the BHJ active layer. The conductive polymer poly(3,4-ethylenedioxythiophene):poly (styrene sulfonic acid) (PEDOT:PSS) dispersed in water is a typical HTL material choice of the conventional device structure [15]. Unfortunately, the hydrophobic nature of the organic material hampers the deposition of the PEDOT:PSS HTL above the BHJ. In addition, the acidic PEDOT:PSS can aggravate the degradation of the BHJ active layer and the electrodes, further affecting device stability [16,17]. For inverted OSCs, the HTL is fabricated more commonly with transition metal oxides, such as molybdenum oxide (MoO_x_), tungsten oxide (WO_x_), and vanadium oxide (VO_x_) [18,19,20]. In order to produce HTLs of good film homogeneity, these metal oxides are usually deposited by thermal evaporation in a high vacuum, which is incompatible with large-scale, roll-to-roll methods.

Nickel oxide (NiO_x_) is a p-type metal oxide with a wide band gap and superior electron-blocking ability. OSCs with NiO_x_ HTLs were reported to outperform the control devices in terms of power conversion efficiency (PCE) and stability [21,22]. Besides thermal evaporation [23], NiO_x_ HTLs can be processed and modified via solution-based methods. Kang et al. developed Cu-doped NiO_x_ as the HTL for PCDTBT:PC_71_BM OSCs. The Cu-doped NiO_x_ enhanced the electrical conductivity of the material and improved the interface contact with the active layer, resulting in a PCE of 7.05%, which was about a 30% improvement over the pristine NiO_x_ HTL device [24]. Kim reported a polyethylene glycol (PEG)-assisted synthesis of the compact NiO_x_ layer as the HTL of PTB7:PC_70_BM OSCs. The uniform and smooth surface morphology of the HTL facilitated better interfacial properties for a more efficient charge transport. As a result, the doping of PEG helped to increase the PCE of NiO_x_-HTL-based devices from 5.68% to 6.91% [25]. It is worth noting that the above examples of solution-processable NiO_x_ HTLs were applied in a conventional device structure via the sol-gel processing technique, which requires a reaction temperature of up to 500 °C to convert the precursor. Such a high temperature is unfavorable for the organic BHJ active layer. Therefore, the sol-gel-based NiO_x_ HTLs are seldom encountered in the inverted device.

Alternatively, constructing the metal oxide HTL by utilizing NP dispersions is considered a more feasible solution-based approach for the inverted-structured OSCs. Nevertheless, in a similar fashion to PEDOT:PSS dispersions, the regulation of active layer wettability for metal oxide NP dispersions remains a problem to be solved that requires extensive trial and error. A few successful attempts at the fabrication of the MoO_x_ NP HTL atop the active layer were previously reported [26,27]. For the NiO_x_ NPs, Margeat et al. deployed isopropanol to address the wettability issue of Cu-doped NiO_x_ NPs. A thin layer of 2,3,5,6-tetrafluoro-7,7,8,8-tetracyanoquino dimethane (F4-TCNQ) was additionally applied to adjust the WF of the HTL for better hole extraction efficiency for the conventional PTB7:PC_70_BM OSC and the inverted PBDB-T-2F (also known as PM6):IT-4F OSC. Despite the Cu-NiO_x (12.2%)_/F4-TCNQ-HTL-based inverted device having an improved performance over the PEDOT:PSS counterpart, the PCE was less than 8% [28]. At present, the application and optimization of the NiO_x_ NP HTL for inverted OSCs are still scarce and need further exploration to achieve satisfying device performance.

Inspired by previous studies [28,29], we developed solution-processable poly(methyl methacrylate) (PMMA)-doped NiO_x_ NP dispersions to fabricate the HTL for the inverted poly[(2,6-(4,8-bis(5-(2-ethylhexyl-3-fluoro)thiophen-2-yl)-benzo[1,2-b:4,5-b’]dithiophene))-alt-(5,5-(1′,3′-di-2-thienyl-5′,7′-bis(2-ethylhexyl)benzo[1′,2′-c:4′,5′-c’]dithiophene-4,8-dione)]:2,2′-((2Z,2′Z)-((12,13-bis(2-ethylhexyl)-3,9-diundecyl-12,13-dihydro-[1,2,5]thiadiazolo[3,4-e]thieno[2”,3″:4′,5′]thieno[2′,3′:4,5]pyrrolo[3,2-g]thieno[2′,3′:4,5]thieno[3,2-b]indole-2,10-diyl)bis(methanylylidene))bis(5,6-difluoro-3-oxo-2,3-dihydro-1H-indene-2,1-diylidene))dimalononitrile (PM6:Y6) BHJ OSCs. PMMA is a commercially available polymer that can be dissolved in aromatic solvents to modify the HTLs of perovskite solar cells for better device performance [30,31,32,33]. In this work, an organic solvent that is miscible with water and alcohol, namely N, N-dimethylformamide (DMF), was adopted to dissolve the PMMA. The dissolved PMMA was subsequently added to the NiO_x_ NP dispersions to investigate its modification effects. As a result, by employing the NiO_x_:PMMA HTL, the inverted OSCs exhibited an optimized power conversion efficiency (PCE) of 15.11%. Additionally, for the device with a PMMA-doped NiO_x_ HTL, the PCE could maintain 75% of its initial value under ambient conditions for an extended period of 30 d.

## 2. Materials and Methods

**Figure 1:**
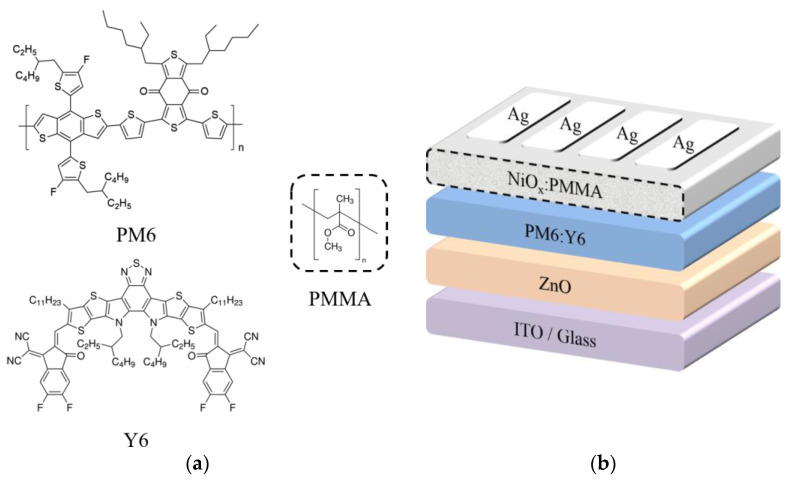
(**a**) Molecular structures of PM6, Y6, and PMMA. (**b**) The device structure of the OSC.

The PM6 donor and Y6 acceptor were purchased from Solarmer Materials, Inc., Beijing, China. The PMMA (*M_w_*~350,000) was purchased from Sigma-Aldrich (Shanghai) Trading Co., Ltd., Shanghai, China. All other chemical reagents were ordered from TCI (Shanghai) Development Co., Ltd., Shanghai, China. The molecular structures of PM6, Y6, and PMMA are displayed in Figure 1a, and the device structure of the OSC is depicted in Figure 1b.

To obtain NiO_x_ NP dispersions, nickel chloride hexahydrate (NiCl_2_·6H_2_O, 2 g, 8.4 mmol) was added to 100 mL ethanol, then stirred at room temperature to obtain a clear green solution. Next, sodium hydroxide (NaOH) solution (5 mol/L) was added to the green solution until the pH value reached 10. The obtained turbid green mixture was centrifuged, and the remaining precipitation was rinsed twice with deionized water and ethanol to remove any soluble impurities. The rinsed product was dried overnight at 120 °C to obtain nickel(II) hydroxide (Ni(OH)_2_) powder. The Ni(OH)_2_ powder was calcined at 300 °C for 2 h to obtain NiO_x_ NPs in black powder. Finally, the NiO_x_ NPs were re-dispersed in the mixture of deionized water, isopropanol, and n-butanol (4:15:1 by volume) via ultrasonic treatment. The NiO_x_ NP concentration was 10 mg/mL.

The PM6:Y6 BHJ blend solution was prepared by stirring the chloroform-dissolved donor and acceptor blend (1:1.2 by weight) with 1-chloronaphthalene as a solvent additive (0.5% by volume) for 24 h, and the blend concentration was 20 mg/mL. Sol-gel precursor of zinc oxide (ZnO) was prepared by dissolving zinc acetate dihydrate (Zn(CH_3_COO)_2_·2H_2_O, 0.1 g) with ethanolamine (NH_2_CH_2_CH_2_OH, 0.029 mL) in 2-methoxy ethanol (CH_3_OCH_2_CH_2_OH, 1.0 mL) after vigorously stirring for 12 h. To obtain the PMMA-doped NiO_x_ NP dispersions, the powder PMMA was dissolved firstly in DMF, and the PMMA concentration was 10 mg/mL. The as-prepared NiO_x_ NP dispersions were subsequently mixed with various contents of PMMA solution (1%, 3%, or 5% v/v) and stirred for 1 h until the mixture was uniformly distributed.

The inverted OSC devices were fabricated with the structure of indium tin oxide(ITO)/ETL/PM6:Y6/HTLs/Ag. The pre-cleaned ITO/glass substrates were treated with UV–ozone for 10 min to improve their wettability with the ETL material. The ZnO precursor was subsequently spin-coated at 5000 rpm for 40 s on the ITO/glass substrate and then annealed at 150 °C for 30 min in the air to form the ETL. In a glove box of pure nitrogen atmosphere, the BHJ blend solution was spin-coated atop the ETL at 2000 rpm for 1 min to fabricate the active layer (100 nm) and then annealed at 100 °C for 10 min. Next, the HTLs (50 nm) were fabricated by spin-coating the undoped or PMMA-doped NiO_x_ NP dispersions at 1000 rpm for 30 s, followed by the 5000 rpm spinning for 5 s to remove the redundant mixed solution for NP dispersing. After drying in the nitrogen-filled glove box for 30 min, the Ag electrodes (100 nm) were sequentially deposited under a high vacuum of 1 × 10^−5^ Pa.

An LED light source (VeraSol-2, Oriel Instruments, WA, USA) was used as the sunlight simulator. Current density–voltage (*J*–*V*) measurements of the OSCs were performed with a semiconductor characterization system (4200A, Keithley, CA, USA). The external quantum efficiency (EQE) studies were performed using a quantum efficiency analysis system (Solar Cell Scan 100, Zolix, Beijing, China). The transmittance spectra of the films were measured using a UV–vis spectrophotometer (UV-3600, Shimadzu, Kyoto, Japan). Ultraviolet photoelectron spectroscopy (UPS) measurements were performed using a photoelectron spectrometer (PHI 5000 VersaProbe III, ULVAC, Chigasaki, Japan). The morphologies of different films were surveyed using an atomic force microscope (MultiMode 8, Bruker, MA, USA) and a field emission scanning electron microscope (GeminiSEM 300, ZEISS, Jena, Germany). The contact angle measurements were performed using Dataphysics OCA200. For the device stability study, all the OSCs were stored without extra encapsulation in the air at 20 °C and 40% relative humidity.

## 3. Results and Discussion

**Figure 2:**
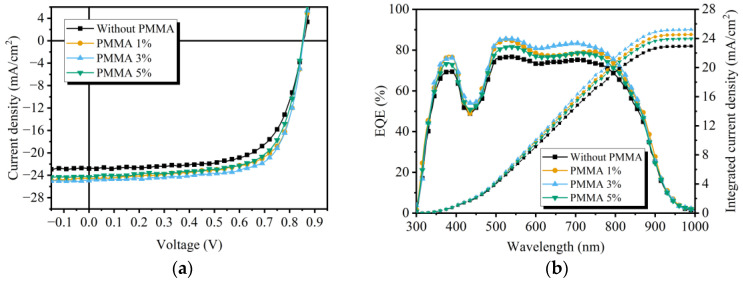
(**a**) *J*–*V* curves of OSCs. (**b**) EQE spectra of OSCs.

To evaluate the photovoltaic performance of inverted PM6:Y6 OSCs with PMMA-doped NiO_x_ NP HTLs, the *J*–*V* curves were measured under AM 1.5G condition. As presented in Figure 2a and Table 1, OSCs with undoped NiO_x_ HTLs exhibit a decent average PCE of 12.86 ± 0.15% with an open circuit voltage (*V*_OC_) of 0.852 V ± 0.007 V, a short-current density (*J*_SC_) of 22.58 mA/cm^2^ ± 0.37 mA/cm^2^, and a fill factor (FF) of 66.71% ± 0.52%. The maximum PCE of the NiO_x_ HTL device is 13.06%. After the doping of PMMA, the devices fabricated with NiO_x_:PMMA HTLs achieve higher average and maximum PCEs compared to the control device. The improvements mainly benefit from the enhanced *J*_SC_ and FF values. By introducing the NiO_x_ NP HTL with 3% of PMMA dopant, the OSC device yields the highest average PCE of 14.95 ± 0.12% and maximum PCE of 15.11%, accompanied by optimized *J*_SC_ of 24.92 mA/cm^2^ ± 0.13 mA/cm^2^ and FF of 70.33% ± 0.45%. As the PMMA content increases to 5%, the *J*_SC_ and the FF exhibit noticeable drops, resulting in declined average and maximum PCEs (14.11 ± 0.10% and 14.25%, respectively), which can be attributed to the less favorable electrical properties of the HTL caused by the excessively doped insulating polymer [31]. It can be anticipated that doping an appropriate amount of PMMA may improve the surface morphology and the deposition quality of the HTL, thus achieving superior device parameters.

To further investigate the enhanced device performance, EQE spectra of OSCs with different HTLs are presented in Figure 2b. After doping PMMA into the NiO_x_ NP HTL, the photoresponse of the modified devices received notable improvements over a broad wavelength range from 300 to 900 nm. The improvements can be ascribed to the more efficient charge carrier transportation and collection. The integrated current density values from the EQE spectra are 22.95 mA/cm^2^, 24.55 mA/cm^2^, 25.22 mA/cm^2^, and 23.97 mA/cm^2^ for devices fabricated with the undoped NiO_x_ HTL and PMMA:NiO_x_ HTLs of different dopant ratios, respectively. The results align with the *J*_SC_ values recorded from the *J*–*V* measurements, indicating their good reliability.

**Figure 3:**
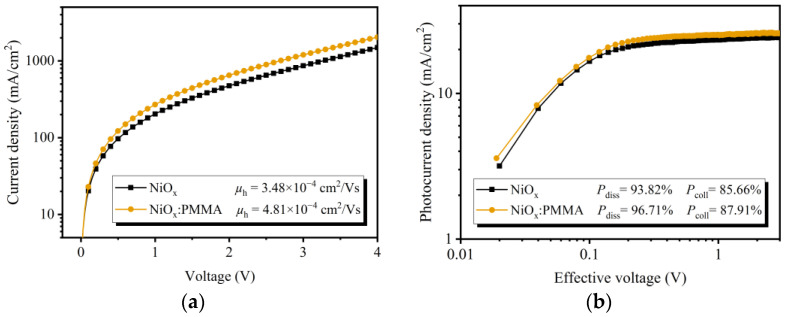
(**a**) *J*–*V* curves of the hole-only devices. (**b**) Photocurrent density dependence on effective voltage.

The hole mobility (*µ*_h_) of the OSCs with different HTLs was evaluated using the space-charge-limited current (SCLC) method [34]. The hole-only devices were fabricated with the structure of ITO/PEDOT:PSS/PM6:Y6/HTLs/ Ag, and the *J*–*V* characteristics in dark conditions are provided in Figure 3a. The device built with the NiO_x_:PMMA HTL provided a higher *µ*_h_ of 4.81 × 10^−4^ cm^2^/Vs than the device of the undoped HTL (*µ*_h_ = 3.48 × 10^−4^ cm^2^/Vs). The enhanced hole mobility indicates that PMMA doping can effectively promote the hole transportation of the NiO_x_ NP HTL.

To gain an in-depth understanding of the exciton dissociation behaviors of the OSC devices, the photocurrent density (*J*_ph_) dependence on the effective voltage (*V*_eff_) was plotted for devices fabricated with NiO_x_ or NiO_x_:PMMA HTLs (Figure 3b) [35]. *J*_ph_ is defined as the difference between illuminated current densities (*J*_L_) and dark current densities (*J*_D_). *V*_eff_ is obtained by the difference between *V*_0_ and *V*_a_, where *V*_0_ is the voltage when *J*_ph_ = 0, and *V*_a_ is the applied bias voltage. The saturated current density (*J*_sat_) can be obtained at a high *V*_eff_ when all excitons are assumed to be dissociated into free charge carriers, and the electrons/holes are collected by the corresponding electrodes. At *V*_eff_ = 3 V, the undoped device has a *J*_sat_ of 23.56 mA/cm^2^, while the device of the NiO_x_:PMMA HTL exhibits a *J*_sat_ of 25.80 mA/cm^2^. Under short-circuit conditions or under maximum power output conditions, respectively, the exciton dissociation probability (*P*_diss_) and the charge collection probability (*P*_coll_) are defined by *J*_ph_/*J*_sat_. The OSC device fabricated with the undoped HTL has a *P*_diss_ of 93.82% and a *P*_coll_ of 85.66%, while the PMMA-doped HTL-based device has a *P*_diss_ of 96.71% and a *P*_coll_ of 87.91%. The result reveals that by introducing the PMMA-doped NiO_x_ NP HTL, the OSC device achieves more efficient exciton dissociation as well as charge collection, which are the leading causes for the improved *J*_SC_.

**Figure 4:**
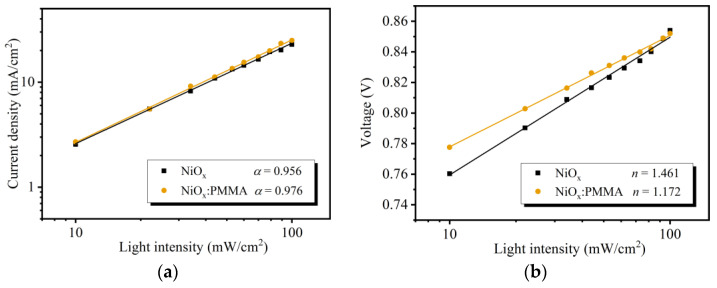
(**a**) *J*_SC_ dependence on incident light intensity (*P*_in_). (**b**) *V*_OC_ dependence on *P*_in_.

As presented in Figure 4a,b, the behaviors of charge carrier recombination were further explored by measuring the dependence of *J*_SC_ or *V*_OC_ on the various incident light intensities (*P*_in_). The degree of bimolecular recombination was qualitatively analyzed by employing the following equation [36]:

*J*_SC_ ∝ *P*_in_^α^ (1)

Under ideal conditions, when the value of α reaches 1, it represents the ideal condition that before recombination occurs, all the free charge carriers are collected at the electrodes. As shown in Figure 4a, the α values of the devices based on the NiO_x_ HTL and NiO_x_:PMMA HTL are 0.956 and 0.976. The slightly higher α value indicates less bimolecular recombination in the PMMA-doped HTL-based OSC device.

The situation of trap-assisted recombination was qualitatively evaluated using the following equation [37]:

*V*_OC_ ∝ *n(kΤ/q)*ln*(P_in_)* (2)

*k* is the Boltzmann constant, *Τ* is the thermodynamic temperature, and *q* is the elementary charge. An *n* value close to 1 suggests trap-assisted recombination occurs less frequently in OSC devices. As depicted in Figure 4b, the *n* value of the OSC device using the undoped NiO_x_ NP HTL is 1.461. For the device with a modified HTL, the *n* value drops significantly to 1.176. The suppressed trap-assisted recombination can account for the enhanced FF in the PMMA-doped device.

**Figure 5:**
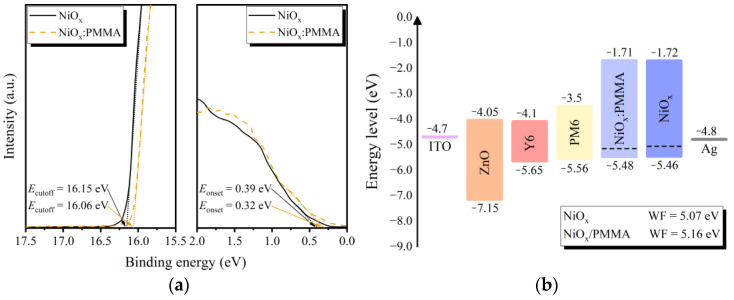
(**a**) UPS spectra of cutoff/onset region for NiO_x_ and NiO_x_:PMMA. (**b**) Schematic diagram of energy level alignments of OSCs.

To study the effect of PMMA doping on energy levels, UPS measurements of pristine and doped NiO_x_ HTLs were conducted. The binding energies of the cutoff region (*E*_cutoff_) and the onset region (*E*_onset_) can be extracted from the UPS spectra in Figure 5a. The band gap energy (*E*_g_) was estimated by the Tauc plot method from the transmittance spectra in Figure S1. The *E*_g_ values for undoped NiO_x_ and PMMA-doped NiO_x_ were 3.74 eV and 3.77 eV, respectively. With the excitation energy value (*hν*) of 21.22 eV (He I), the WF was calculated by subtracting the *E*_cutoff_ from the *hν*. The pristine NiO_x_ HTL had a WF of 5.07 eV. For the NiO_x_:PMMA HTL, the WF value increased to 5.16 eV. Combining the results of *E*_g_, WF, and *E*_onset_, the valence band maximum (VBM) and conduction band minimum (CBM) can be calculated. For the pristine and modified HTLs, the VBM levels were 1.72 eV and 1.71 eV, and the corresponding CBM levels were 5.46 eV and 5.48 eV, respectively. Given the above results, the energy level diagram of the OSCs is plotted in Figure 5b. Rather than the recombinational hole transport of other metal oxides (e.g., MoO_x_) that occurs at the interface between the active layer and HTL, NiO_x_ exhibits intrinsic p-type hole transport, whereby the photogenerated holes from the donor are directly transported to the anode through the valence band of the HTL [29]. Therefore, the energy level matching between the WF of the NiO_x_ HTL and the HOMO of the donor is an important factor for achieving desirable device performance [38]. The deeper WF and the shifted energy level alignments of the PMMA-doped NiO_x_ HTL help to optimize the hole extraction and collection efficiencies, thus contributing to the improved *J*_SC_ and FF for the modified OSC device.

**Figure 6:**
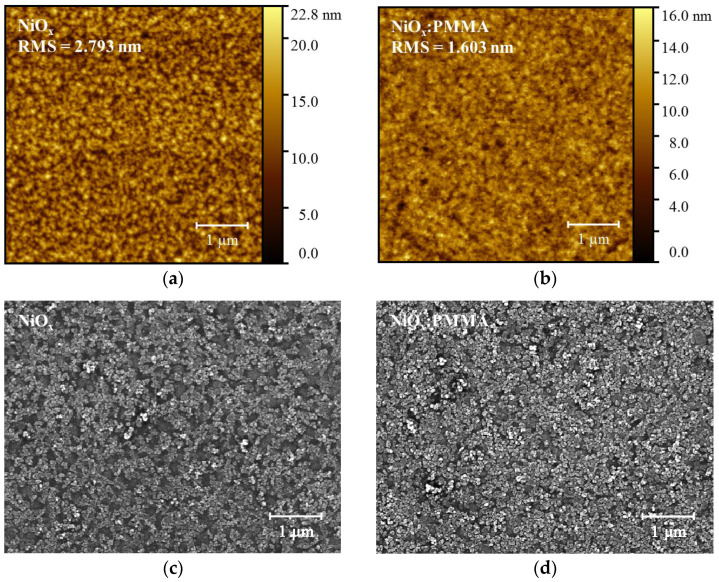
(**a**) Atomic force microscopy (AFM) height image of NiO_x_ HTL film. (**b**) AFM height image of NiO_x_:PMMA HTL film. (**c**) Top-view scanning electron microscopy (SEM) image of NiO_x_ HTL film. (**d**) Top-view SEM image of NiO_x_:PMMA HTL film.

To reveal the morphological evolutions of the NiO_x_ NP HTL after the modification of PMMA, the surface morphologies of the different HTLs were characterized by atomic force microscopy (AFM) and scanning electron microscopy (SEM). The AFM height images of the bare NiO_x_ HTL and the PMMA-doped NiO_x_ HTL are shown in Figure 6a,b. The bare NiO_x_ HTL has a relatively coarse surface, accompanied by a root mean square (RMS) roughness value of 2.793 nm. By contrast, the PMMA-doped HTL has a smoother surface with a significantly decreased RMS value of 1.603 nm. From the top-view SEM images in Figure 6c,d, visible pinholes can be found for the unmodified NiO_x_ HTL. After the doping of PMMA, the long-chain polymer with high molecular weight could fill the gaps between the NiO_x_ NPs, resulting in an HTL film of more continuously organized NPs with fewer pinholes [39,40]. The AFM and SEM results suggest improved surface smoothness and uniformity of the NiO_x_:PMMA HTL film, which could be beneficial for promoting charge transportation and restricting charge recombination. The pinhole-less and denser morphology of the PMMA-doped HTL could also help to improve device stability by resisting the penetration of water, oxygen, and the top metal electrode [33].

**Figure 7:**
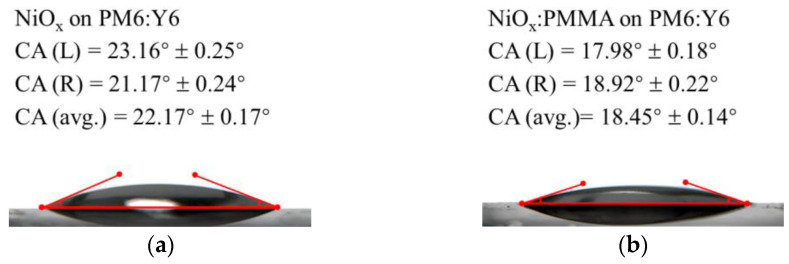

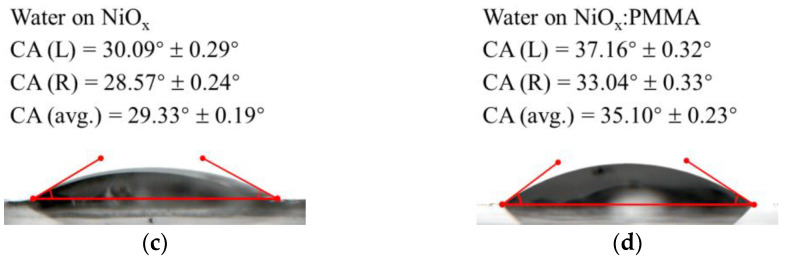
(**a**) Contact angle (CA) image of NiO_x_ NP dispersions on PM6:Y6 film, (**b**) CA image of NiO_x_:PMMA NP dispersions on PM6:Y6 film, (**c**) CA image of water on NiO_x_ HTL film, and (**d**) CA image of water on NiO_x_:PMMA HTL film. Each CA value was obtained from 5 attempts of measurement.

Contact angle (CA) measurements were carried out to collect additional evidence of improvements brought about by doping the PMMA. The CA images of undoped or PMMA-doped NiO_x_ NP dispersions on the PM6:Y6 BHJ film are provided in Figure 7a,b. Owing to the incorporation of the polymer, the modified NiO_x_ NP dispersions have a reduced CA from 22.17° ± 0.17° to 18.45° ± 0.14°, indicating its improved wettability on the BHJ. This could benefit the deposition quality of the HTL film, further boosting the device performance of OSCs [29]. The CA images of distilled water on the NiO_x_ and NiO_x_:PMMA HTL films are displayed in Figure 7c,d. Since the CA of water on the NiO_x_:PMMA film increased from 29.33° ± 0.19° to 35.10° ± 0.23° over the pristine film, the enhanced hydrophobicity of the HTL could provide better protection for the active layer from water in ambient conditions.

**Figure 8:**
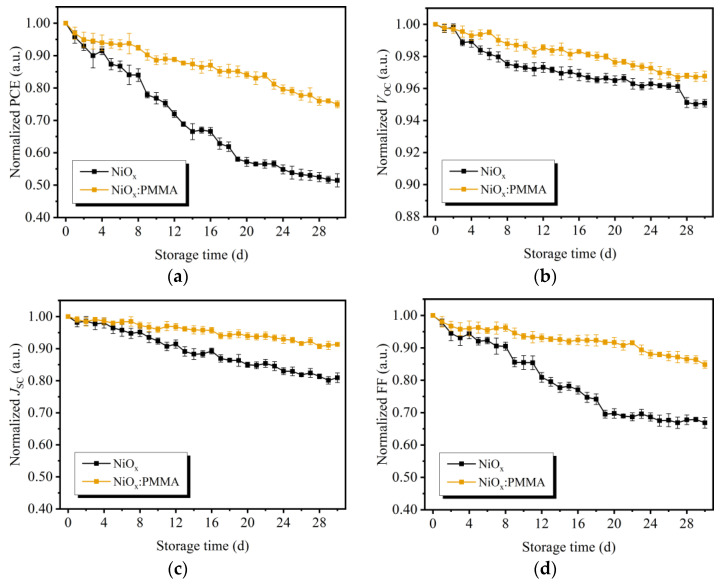
Normalized device performance parameters of OSCs after storing in ambient conditions for 30 d (statistical values obtained from 10 undoped or PMMA-doped devices fabricated in parallel): (**a**) PCE, (**b**) *V*_OC_, (**c**) *J*_SC_, and (**d**) FF.

Device stability is a crucial factor for the commercial applications of OSCs. To investigate the long-term effects of PMMA doping, the OSCs with undoped or PMMA-doped NiO_x_ HTLs were stored without extra encapsulation in ambient conditions for 30 d. During this period, *J*–*V* measurements were performed daily to study the evolutions of device performance parameters. As seen in Figure 8, after being stored in air for 30 d, both the devices with undoped or PMMA-doped HTLs were able to maintain over 95% of their initial *V*_OC_ values. However, the undoped device suffered notable degradations in *J*_SC_ and FF. Consequently, its PCE decreased to 51% of the initial values. In comparison, the *J*_SC_ and FF of the PMMA-doped device declined less significantly, resulting in a PCE of 75% of the initial value. The improved device performance stability in ambient conditions can be attributed to the enhanced moisture/oxygen-blocking effects of the NiO_x_:PMMA HTL, which is in accordance with the results of morphology and CA characterizations.

## 4. Conclusions

In this study, a facile modification strategy of a solution-processable NiO_x_ NP HTL was realized for the inverted-structured OSCs. By doping the NiO_x_ NP dispersions with a commercially available polymer, namely PMMA, the PM6:Y6 device with the modified HTL exhibited an average PCE of 14.95 ± 0.12% and a maximum PCE of 15.11%, which was a significant improvement from the PCEs of the undoped-NiO_x_-HTL-based device. With more favorable electrical and surface properties of the NiO_x_:PMMA HTL, efficient charge transportation as well as suppressed charge recombination were simultaneously accomplished. The PMMA-doped HTL also helped the OSC device to maintain 75% of its initial PCE value after aging in ambient conditions for 30 d. Overall, this work proposed a novel solution-processable, polymer-assisted metal oxide nanocomposite HTL for the future mass production of inverted OSCs with excellent device performance and prolonged device shelf life [41].

## Figures and Tables

**Table 1:** Photovoltaic performance parameters of OSCs with pristine NiO_x_ HTLs and NiO_x_:PMMA HTLs with different PMMA doping ratios.

PMMA Doping Ratio	*V*_OC_ (avg.) (V)	*J*_SC_ (avg.) (mA/cm^2^)	FF (avg.) (%)	PCE (avg.) ^1^ (%)	Max. PCE (%)
Without PMMA	0.852 ± 0.007	22.58 ± 0.37	66.71 ± 0.52	12.86 ± 0.15	13.06
PMMA 1%	0.852 ± 0.004	24.35 ± 0.28	69.01 ± 0.77	14.32 ± 0.18	14.51
PMMA 3%	0.853 ± 0.006	24.92 ± 0.13	70.33 ± 0.45	14.95 ± 0.12	15.11
PMMA 5%	0.851 ± 0.011	24.11 ± 0.22	68.76 ± 0.88	14.11 ± 0.10	14.25

^1^ The average values were obtained from 20 devices fabricated in parallel.

## Data Availability

The data presented in this study are available on request from the corresponding author.

## Supplementary Materials

The following supporting information can be downloaded at: https://www.mdpi.com/article/10.3390/polym15081875/s1, Figure S1. (a) Transmittance spectra of ITO, ITO/NiO_x_, and ITO/NiO_x_:PMMA films. (b) Tauc plots of NiO_x_ and NiO_x_:PMMA films.
