# Supplementary material for: Solution-Processable NiOx:PMMA Hole Transport Layer for Efficient and Stable Inverted Organic Solar Cells
Source: Polymers (Basel). 2023 Apr 14;15(8):1875. doi: 10.3390/polym15081875 (PMC10144863; doi:10.3390/polym15081875)
Supplement: Supplementary file 1 [file polymers-15-01875-s001.zip › polymers-2288924-supplementary.pdf]

Supplementary Materials

Solution-Processable NiO_x:PMMA Hole Transport Layer for Efficient and Stable Inverted Organic Solar Cells

Tianyu Kong [1], Genjie Yang [1], Pu Fan [1,*], and Junsheng Yu [1,*]

[1] State Key Laboratory of Electronic Thin Films and Integrated Devices, School of Optoelectronic Science and Engineering, University of Electronic Science and Technology of China, Chengdu 610054, China; ty_kong@std.uestc.edu.cn (T.K.); genjieyang@std.uestc.edu.cn (G.Y.)

* Correspondence: fan.pu@hotmail.com (P.F.); jsyu@uestc.edu.cn (J.Y.)

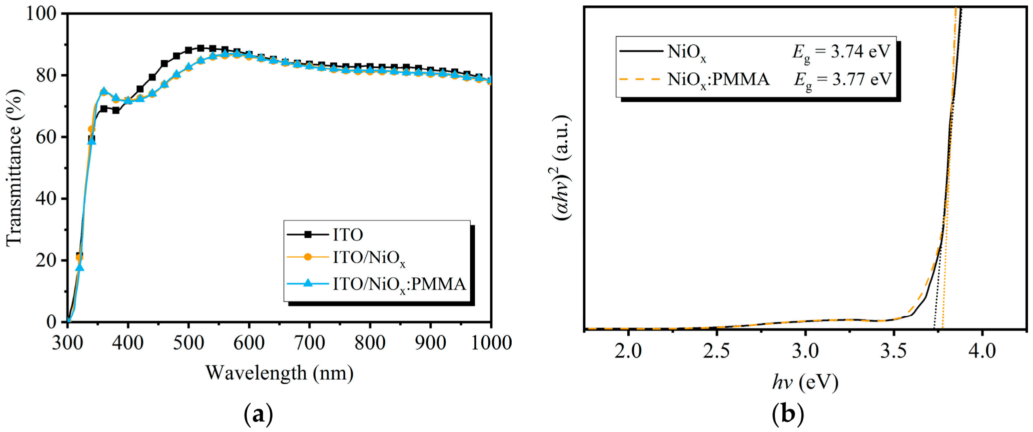

Figure S1. (**a**) Transmittance spectra of ITO, ITO/NiO_x, and ITO/NiO_x:PMMA films. (**b**) Tauc plots of NiO_x and NiO_x:PMMA films.
